# Natural killer cells expanded on membrane-bound IL15 or IL21 activate different miRNA transcriptional profiles with functional consequences

**DOI:** 10.1186/2051-1426-3-S2-P288

**Published:** 2015-11-04

**Authors:** Anitha Somanchi, Srinivas Somanchi, Dean Lee

**Affiliations:** 1 UT MD Anderson Cancer Center, Houston, TX, USA

## 

MicroRNA (miRNA) are non-coding regulatory RNA that are key posttranscriptional regulatory machinery of cells. miRNA play important role in several physiological functions during embryonic development, differentiation, cancer progression as well as hematopoiesis, and regulation of immune responses. miRNA regulate development of various immune cells including T and Natural killer (NK) cells. Owing to significant interest in adoptive immunotherapy, numerous methods for expanding NK cells have been developed in recent years. We have previously developed a robust platform for ex-vivo expansion of human NK cells using K562 expressing membrane-bound IL21 (K562.mbIL21). The study of the role of miRNA in NK cells biology is an emerging area of research in development, homeostasis and immune responses. However, the role and effect of expansion of NK cells on miRNA and consequent impact on post-transcriptional regulation of protein expression in expanded NK cells remain to be studied.

In this study we investigated the role of distinctive miRNA expression profiles that could influence post-transcriptional regulation of gene expression, and consequently differential protein expression in NK cells expanded on K562.mbIL21 platform compared to primary unexpanded NK cells. We had previously shown that K562.mbIL21 platform enables significantly higher expansion of NK cells compared to K562 expressing membrane bound IL15 (K562 mbIL15), therefore in this study we also compared miRNA expression profiles of NK cells expanded on K562.mbIL15 and K562.mbIL21. We used primary protein and miRNA obtained from, human NK cells derived from 4 healthy donors, Pre and post-expansion on K562.mbIL15 and K562.mbIL21 platforms. miRNA expression profiles were analyzed using Nanostring's nCounter. Initial screening of our dataset revealed 13 miRNAs that are differentially expressed by at least 16 folds between fresh and expanded NK cells or mbIL15 and mbIL21. Comparison between the mbIL15 and mbIL21 expanded cells revealed differential expression of miRNAs critical for regulation of cell cycle proteins as well as key cytokines (TNF-a and interferon-g) such as miR-124-3p and miR 146a-5p (76x and 32x lower in mbIL21 expanded cells respectively). Also, analysis of protein expression by RPPA revealed upregulation of cell cycle proteins cyclin D1 and AMPK, and downregulation of pro-apoptotic protein Bim in mbIL21 expanded NK cells compared with mbIL15 and/or primary unexpanded cells. We are currently correlating miRNA expression and proteomic profiles of expanded and unexpanded cells to identify possible targets of miRNA regulation and subsequently study specific roles of these miRNA in this expansion system by overexpression o/blockade of target miRNA.

